# Reducing malnutrition in critically ill pediatric patients

**DOI:** 10.5935/0103-507X.20180034

**Published:** 2018

**Authors:** Caroline Abud Drumond Costa, Pedro Celiny Ramos Garcia, Daiane Drescher Cabral, Cristian Tedesco Tonial, Francisco Bruno, Paulo Roberto Enloft, Kelly Daiane Stochero Velozo

**Affiliations:** 1 Programa de Pós-Graduação em Pediatria e Saúde da Criança, Pontifícia Universidade Católica do Rio Grande do Sul - Porto Alegre (RS), Brasil.; 2 Unidade de Terapia Intensiva Pediátrica, Hospital São Lucas, Pontifícia Universidade Católica do Rio Grande do Sul - Porto Alegre (RS), Brasil.; 3 Pontifícia Universidade Católica do Rio Grande do Sul - Porto Alegre (RS), Brasil.

**Keywords:** Nutritional status, Pediatric intensive care unit, Malnutrition, Mortality, Prognosis

## Abstract

**Objective:**

To compare the prevalence of malnutrition during two time periods in a pediatric intensive care unit.

**Methods:**

This was a retrospective cross-sectional study of patients admitted to the pediatric intensive care unit of a university hospital during two one-year periods with an interval of four years between them. Nutritional evaluation was conducted based on weight and height measured at admission. The body mass index-for-age was the parameter chosen to evaluate nutritional status, as classified according to the World Health Organization, according to age group.

**Results:**

The total sample size was 881 (406 in the contemporary sample and 475 in the historical sample). There was a significant reduction in malnutrition in the contemporary sample (p = 0.03). Malnourishment in patients in the historical sample was significantly associated with mortality and length of stay, while malnourishment in patients in the contemporary sample was not associated with worse outcomes.

**Conclusion:**

There was a significant reduction in malnutrition among patients in the same pediatric intensive care unit when comparing the two time periods. Our findings of a change in nutritional profile in critically ill patients corroborate the nutritional status data of children and adolescents worldwide.

## INTRODUCTION

Anthropometric evaluation is a necessary tool for monitoring children's health and is an important item when estimating a child's nutritional status and monitoring her growth and development.^(^^1^^)^ In a hospital setting, the evaluation helps to identify eating disorders, supports diagnosis and facilitates prognosis, making early and safe interventions possible.^(^^2^^-^^5^^)^

Studies conducted in the hospital environment have identified a high prevalence of malnutrition on admission.^(^^5^^-^^7^^)^ In intensive care units (ICU), the nutritional status of patients tends to deteriorate due to obstacles that hinder the provision of ideal nutrition, such as volume restrictions, procedures and interventions, disease severity, frequent food breaks and lack of standardization of evidence-based processes for better nourishment.^(^^2^^,^^7^^,^^8^^)^

Another important aspect to consider is the opposite of inadequate nutritional status-obesity, which is considered to be a public health problem by the World Health Organization (WHO), and it affects an alarming and growing proportion of children and adolescents around the world. Due to its comorbidities and complications, obesity can jeopardize the population's longevity.^(^^9^^)^

Speculation in regard to modifying the nutritional profiles of patients admitted to hospital institutions has been observed among researchers in the field, such as in the study by Castro et al. in a pediatric ICU (PICU) in Mexico, which identified a reduction in malnutrition and significant increases in excess weight and obesity in children upon admission.^(^^10^^)^

Patients in a PICU with nutritional status inadequacies can experience unfavorable outcomes (i.e., mechanical ventilation - MV, mortality, longer length of stay and infection); therefore, it is extremely important to assess the unit's nutritional profile.^(^^11^^)^

Our goal was to compare the prevalence of malnutrition in a PICU during two time periods and to evaluate its relationship with severity and the following outcomes: need for MV, length of stay and mortality.

## METHODS

This was a cross-sectional retrospective study. The sample consisted of patients admitted in two one-year periods, with an interval of four years, in the PICU of a university hospital in southern Brazil. This unit is characterized as mixed and receives medical and surgical patients from the emergency ward, infirmary, surgery and other hospitals. It has 12 beds.

For comparison, we named the two time periods as follows: the historical sample, consisting of patients who were admitted from September 2009 to August 2010, and the contemporary sample, consisting of patients who were admitted in the period between June 2013 and June 2014.

The exclusion criteria used were based on Pollack et al.'s 1988 study^(^^12^^)^ and were as follows: length of stay in the unit of less than 8 hours, patients for whom anthropometric measurements were not taken and patients whose length of stay exceeded 90 days.

Demographic data were collected upon patient admission and were recorded in the unit's database, as is typical for patient admission and discharge. This database is routinely reviewed by the unit's research team.

Patients were classified as medical or surgical; in terms of origin, they were classified as internal (coming from the surgical block or infirmary) or external (coming from emergency or from outside the hospital and transferred directly to the PICU).

Anthropometric measurements (weight and height) were taken by the nursing team at admission and were transferred to the WHO Anthro 3.1.0 (zero to 5 years of age) and WHO AnthroPlus 1.0.2 (age 5 to 19) software programs, which were used to perform the analysis of each individual's nutritional status. Data are expressed as a Z score, and WHO curves were used as the reference standard.^(^^13^^,^^14^^)^ We chose body mass index-for-age (BMI/A) as the malnutrition indicator for our main comparison, as it applies to all age groups. The cutoff points used followed the Nutritional Status Technical Classification Standard of the Food and Nutrition Surveillance System (Sistema de Vigilância Alimentar e Nutricional - SISVAN) for children and adolescents.^(^^15^^)^ For stratification purposes, nutritional status classification was subdivided as follows: BMI/A, into Z < -2 for malnourished (thinness and severe wasting) and Z ≥ -2 for non-malnourished (eutrophy, risk of excess weight, overweight, obesity and severe obesity).

The Pediatric Index of Mortality (PIM 2)^(^^16^^)^ was used to evaluate disease severity. Score calculations were performed by the unit's doctors. The results were stratified into PIM 2 above or below 6% (PIM 2 < 6 and PIM 2 > 6), considering patients with PIM 2 > 6% to be more severe, taking into account the unit's historical mortality.

Patients were classified according to their main organ dysfunction at admission (respiratory, neurological, cardiac, hematologic, hepatic and renal). We also evaluated the presence of these disorders during hospitalization. Multiple organ dysfunction syndrome (MODS) was determined to be simultaneous dysfunction episodes in two or more organs. The definition of organ dysfunction was adapted from the International Pediatric Sepsis Consensus Conference, 2005, published by Goldstein et al.^(^^17^^)^

The main outcomes were the need for MV, length of stay and mortality in both study periods. The cause of discharge was either clinical improvement or death. The difference in days from admission to discharge or death was used to evaluate the length of stay. For the outcome analysis, we stratified the length of stay variable into prolonged (length of stay ≥ 7 days) and not prolonged (length of stay < 7 days).

Numerical data were expressed as absolute and percentage values. The descriptive analysis included the mean, standard deviation, median and interquartile range. Pearson's chi-square test was used to evaluate the association between nutritional status and outcomes, and Fisher's exact test was used when the expected frequency was ≤ 5. The Mann-Whitney test was used for the continuous variables that characterized the sample (asymmetric distribution). P values of < 0.05 were considered statistically significant. Data analysis was performed using the IBM Statistical Package for the Social Sciences program (IBM SPSS Statistics 17.0).

This study was approved by the institutional research ethics committee (protocol number 790,498) on September 12, 2014. The researchers signed terms of agreement for data usage.

## RESULTS

**Figure 1:**
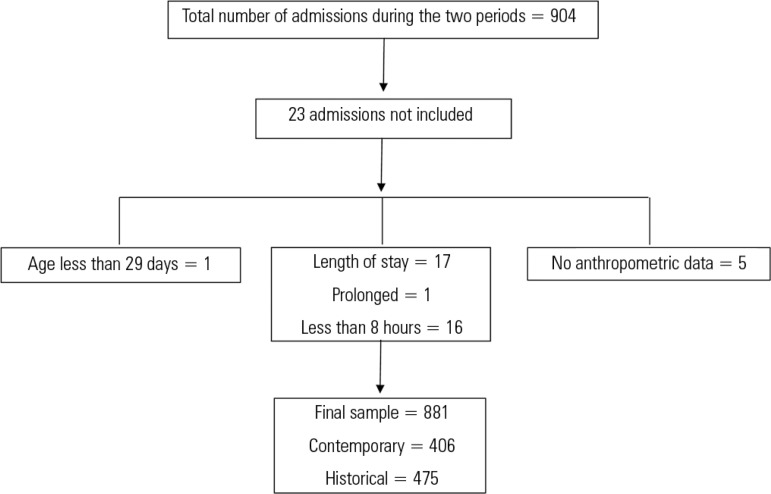
Patient inclusion flowchart.

The total number of admissions in the two periods was 904 patients. Twenty-three patients were not included in the analysis due to the exclusion criteria. Thus, 881 admissions were analyzed. Of these, 406 patients were included in the contemporary sample, and 475 were included in the historical sample. The patient inclusion flowchart is shown in figure 1.

We observed that the samples were similar with respect to most of their admission characteristics, with a significant difference only for patient type, with a significant reduction in malnutrition, according to the BMI/A, and a reduction in patient admissions classified as clinical. The admission data are shown in table 1.

**Table 1:** Comparison of admission characteristics by sample

	Contemporary sample n = 406	Historical sample n = 475	p value
Male	221 (54.4)	284 (59.8)	0.109
Age (months)	19.5 (5 - 77)	21.5 (6 - 75)	0.533
Weight (kg)	9.6 (6.3-22)	10.9 (6.5 - 20)	0.696
Height (cm)	79.5 (63 - 115)	82 (64 - 114)	0.448
Malnutrition according to BMI/A	64 (15.8)	101 (21.3)	0.037*
PIM 2	14 (4-38)	11 (4-34)	0.226
Clinical patient	232 (57.1)	303 (63.8)	0.044*

BMI/A - body mass index-for-age; PIM - Pediatric Index of Mortality.

* Significant difference. The results are expressed as n (%) and medians (interquartile ranges).

In the analysis of outcomes between malnourished and non-malnourished patients, in the historic sample, malnutrition was significantly associated with death and length of stay, whereas in contemporary sample, there were no associations between malnutrition and any of the evaluated outcomes.

In regard to severity (PIM 2 > 6), malnourished patients in the historical sample exhibited a more severe condition at admission than did the non-malnourished patients (p = 0.02), whereas in the contemporary sample, the malnourished patients did not differ from the non-malnourished patients in terms of severity (Table 2).

**Table 2:** Comparison between malnourished and non-malnourished patients by sample

Outcome	Contemporary sample n = 406			Historical sample n = 475		
	Malnourished n = 64 n (%)	Non-malnourished n = 342 n (%)	p value	Malnourished n = 101 **n (%)**	Non-malnourished n = 374 n (%)	p value
PIM 2 > 6	14 (21.9)	72 (21.1)	0.883	25 (24.8)	56 (15)	0.020*
MV	30 (46.9)	163 (47.7)	0.908	51 (50.5)	156 (41.7)	0.114
LS > 7 days	20 (31.3)	96 (28.1)	0.605	35 (34.7)	90 (24.1)	0.032*
MODS	31 (48.4)	126 (36.8)	0.804	55 (54.5)	166 (44.4)	0.072
Death	2 (3.1)	13 (3.8)	0.792	13 (12.9)	10 (2.7)	<0.001*

PIM - Pediatric Index of Mortality; MV - mechanical ventilation; LS - length of stay; MODS - multiple organ dysfunction syndrome.

* Significant difference.

## DISCUSSION

The results of this study show that there was a change in the nutritional profile of critical pediatric patients in the studied unit, with a significant reduction in malnutrition at the time of admission.

With regard to general characteristics upon admission, the unit maintained the same profile when the two samples were compared, as was found in previous studies (2002 and 2010).^(^^18^^,^^19^^)^ When evaluating the main reason for admission, we also observed similarity between the samples. The most prevalent organ dysfunction during the two time periods was respiratory, followed by neurological. These characteristics are common in the PICU, as reported in other studies.^(^^20^^,^^21^^)^

When we compared the severity and outcomes of malnourished patients between the two time periods, the malnourished patients in the historical sample experienced more severe conditions and worse outcomes. In our contemporary sample of patients, malnutrition was not related to any of the outcomes measured (length of stay > 7, MV, MODS and mortality). This result can be explained by the relationship between severity data and outcomes, as in the contemporary sample, patient malnourishment was less severe upon admission. We can also speculate that these data may have been affected by our stratification because patients who we considered "not malnourished" were those with Z > -2 BMI/A, i.e., those who exhibited a normal weight, risk of excess weight, overweight, obesity and severe obesity. We did not evaluate how the number of patients who were overweight and obese may have affected the outcomes.

There are many studies evaluating the relationship between nutritional status and severity and outcomes in the PICU, but these studies are heterogeneous in terms of methodology and results. Malnutrition has an independent association with MV time but is not a predictor of mortality.^(^^22^^)^ In a study conducted in a PICU in Brazil, Zamberlan et al. evaluated patients after liver transplantation and found no association between nutritional status and mortality but did find an association between nutritional status and length of stay.^(^^23^^)^ Delgado et al. observed that malnourished patients had a worse inflammatory response, but they did not find an association with outcomes.^(^^24^^)^ Mota et al., in turn, studied the effect of malnutrition on MV usage and found that malnourished patients required more MV and remained hospitalized for a longer time.^(^^25^^)^

In the analysis conducted in our study, the reduction of malnutrition at admission was 6% over the four-year period. The percentage of malnourished patients found is notable when compared with a study conducted by our research group, in which Einloft et al. evaluated the epidemiological profile of 16 years of the PICU in a sample of 13,131 patients and found that 30% of patients exhibited malnutrition on admission.^(^^18^^)^ These data suggest that the nutritional transition is also reflected in the hospital environment.

Studies conducted in different countries have shown similar findings to those obtained previously by our group, with the percentage of malnourished patients varying between 20% and 50%.^(^^7^^,^^26^^)^ In the present study, the percentage of malnourished patients in the contemporary sample was 15.8%, i.e., lower than that reported in the above-mentioned studies and one of the lowest figures encountered. Our results are close to those of a multicenter study by Mehta et al., who evaluated the nutritional status of patients from 31 PICUs in eight countries and found that 17.1% of patients were malnourished. This finding was according to a BMI score of (Z < -2), the same parameter and stratification as used in our study.^(^^27^^)^

Some authors have noted the need to study the possible complications of overweight and obese critical pediatric patients with unfavorable outcomes in the PICU.^(^^28^^-^^30^^)^ This issue remains unclear, and the relationships between this change in nutritional profile and negative outcomes should be investigated.

It is important to note that the data available in regard to the relationship between nutritional status and clinical outcomes, such as those used in the present study, have originated from observational studies; therefore, it is difficult to draw conclusions due to the heterogeneity and limitations of those studies.^(^^11^^)^

Some limitations of our study should be considered: the measurement of anthropometric data is likely to be impaired at the time of admission due to patient instability; BMI/A alone was used to define nutritional status, and other variables were not taken into account; we did not evaluate nutritional development in this population but rather the nutritional profile, on both occasions, evaluated at admission time.

## CONCLUSION

By studying two periods with a four-year interval in the same PICU, we were able to observe a change in the unit's nutritional profile. We observed a significant decrease in malnutrition according to BMI/A across this time period, and in the contemporary sample, we found no differences between malnourished and non-malnourished patients in terms of the evaluated outcomes. The data in our sample corroborate data relating to nutritional profile changes already observed among children and adolescents in the populations of most countries in which malnutrition is becoming an increasingly less common condition.
